# County Veterinary Medical Society of the State of New York

**Published:** 1882-04

**Authors:** 


### COUNTY VETERINARY MEDICAL SOCIETY OF THE STATE OF NEW YORK.

The County Veterinary Medical Society was incorporated in January, 1879, under the General Laws of this State governing such institutions. The promoters of this Society contended that there were no opportunities given to practical veterinary surgeons to prove their capabilities; and after organizing, they appointed a Board of Censors to examine any person who has been in actual veterinary practice for five years, and, if found competent, a certificate of qualification was granted.

The first year, twenty-seven applicants appeared for examination, and only four were given certificates; in the second year, twenty-three, and only five were given.

The Society has since increased in membership to fifty-six; includes veterinary surgeons from all over the State, constituting it the largest veterinary organization in the United States. They hold their meetings on the first Saturday in each month, in Room 24, Cooper Institute, at eight o'clock in the evening. The Society, at its meetings, debate over the diseases at the time prevalent, and obtain the various opinions and line of treatment adopted by each, with the result. Papers are read on various scientific topics appertaining to dumb animals, communications are received from the rural districts, giving the opinion of the country veterinary surgeons on the prevailing diseases in their localities. Their meetings are very interesting, and the Society promises to be a large and flourishing veterinary organization. They had an annual meeting in March, and elected as president, Dr. W. T. Carmody, M.R.C.V.S.; vice-president, Prof. J. A. Going, M.R.C.V.S.; secretary, G. P. Delisser, V.S.; assistant secretary, E. L. Tavanes, V.S.; treasurer, W. C. Bretherton, V.S. The Board of Censors for the ensuing year are: Professor C. I. Smith, M.D.; Prof. R. W. Finlay, D.V.S.; Prof. J. A. Going, M.R.C.V.S.; R. A. Finlay, D.V.S.; Ralph Ogle, V.S.

